# Transcriptional responses in different mouse models of septic liver injury differ from those in patients with septic liver injury

**DOI:** 10.3389/fimmu.2025.1556392

**Published:** 2025-07-30

**Authors:** Qin Yan, Wei Fan, Xinsen He, Shi Zheng, Xiaolin Zhong

**Affiliations:** ^1^ Department of Gastroenterology, The Affiliated Hospital of Southwest Medical University, Luzhou, China; ^2^ The Public Platform of Advanced Detecting Instruments, Public Center of Experimental Technology, Southwest Medical University, Luzhou, China; ^3^ Human Microecology and Precision Diagnosis and Treatment of Luzhou Key Laboratory, Luzhou, China; ^4^ Metabolic Vascular Diseases Key Laboratory of Sichuan Province, Department of Cardiovascular Surgery, The Affiliated Hospital, Southwest Medical University, Luzhou, Sichuan, China; ^5^ Key Laboratory of Cardiovascular Remodeling and Dysfunction, Department of Cardiovascular Surgery, The Affiliated Hospital, Southwest Medical University, Luzhou, Sichuan, China

**Keywords:** SLI, cecal ligation model, lipopolysaccharide, immune infiltration, *SOCS3*, microarray

## Abstract

**Introduction:**

Sepsis, particularly sepsis-induced liver injury (SLI), exhibits acute onset and high mortality (up to 80%). While murine models are widely used for mechanistic studies due to limited human sample availability, their accuracy in replicating human SLI pathophysiology remains debated.

**Methods:**

Human SLI transcriptomes were characterized to identify core genes and immune signatures using Venn analysis and immune infiltration profiling. Transcriptomic features of two murine SLI models—cecal ligation and puncture (CLP) and lipopolysaccharide (LPS) challenge—were benchmarked against human SLI to evaluate pathophysiological relevance. Both models were then utilized to validate core gene expression for SLI biomarker identification.

**Results:**

Human SLI transcriptomics revealed significant enrichment in apoptotic processes, NF-κB regulation, inflammatory responses, protein phosphorylation, and bacterial response. Key pathways included IL-17 signaling, antigen processing, estrogen signaling, and atherosclerosis. Immune infiltration confirmed multifactorial immune cell involvement. Both murine models recapitulated inflammatory and immune responses, with the LPS model mimicking human SLI via chemotaxis, phagocytosis, NOD-like receptor signaling, and leukocyte migration. The CLP model uniquely replicated neutrophil chemotaxis, apoptosis, ER stress, IL-17, and TNF signaling. SOCS3 was validated as a potential SLI biomarker.

**Discussion:**

Murine models partially replicate human SLI pathology but exhibit distinct mechanistic emphases. Careful model selection is essential for biomarker discovery (e.g., SOCS3) and pathogenic mechanism exploration, highlighting inherent model limitations.

## 1 Introduction

Sepsis is a common critical illness occurring in Intensive care units (ICUs) and is defined as a life-threatening organ dysfunction syndrome caused by dysregulation of the host response to infection (1, 2). The liver, a target organ affected by sepsis, is a significant contributor to patient mortality (3–5). The liver is involved in various pathophysiological processes during sepsis, including inflammatory and immune responses, oxidative phosphorylation, and cell death (6–8); Due to the scarcity of human samples, numerous studies have used the CLP and LPS models to investigate the mechanisms. The pathogenic mechanism of these disease models’ applicability to human disease treatment requires further clarification. Moreover, because of the acute onset and severe symptoms of sepsis, many studies have focused on identifying diagnostic markers for early diagnosis and prognosis. However, the underlying mechanisms of these markers have been identified in only a few studies, indicating a need for further research.

Microarray technology and bioinformatics analyses play important roles in identifying disease characteristics and biomarkers. By analyzing microarray data from disease models, we used bioinformatics methods to identify key molecular targets and signaling pathways, thereby providing new insights into the pathogenesis, diagnosis, and treatment of the disease (9, 10). In this study, we analyzed the GSE63311 and GSE137340 datasets obtained from the Gene Expression Omnibus (GEO) database and identified 77 DEGs. We explored the potential pathogenesis of SLI through Gene Ontology (GO) functional analysis and Kyoto Encyclopedia of Genes and Genomes (KEGG) pathway enrichment. Eleven core genes were identified through protein–protein interaction (PPI) network analysis and Cytoscape. To validate the disease model’s consistency both *in vivo* and *in vitro*, we further elucidated the molecular mechanisms and pathways associated with DEGs in SLI. We conducted GO functional analysis and KEGG pathway enrichment analysis using the GSE184167 and GSE166488 mouse SLI datasets. In addition, we performed an immune infiltration correlation analysis on GSE137340, which revealed a significant association between sepsis and the immune response. Finally, we established SLI models using the CLP method and intraperitoneal injection of LPS. qRT–PCR validation indicated that *SOCS3* may serve as a potential biomarker for liver injury in sepsis.

## 2 Methods

### 2.1 Microarray data

GEO (https://www.ncbi.nlm.nih.gov/geo/) is an international public data repository (11). We downloaded four gene expression datasets from the GEO database for this study: GSE63311 (12), GSE137340, GSE184167 (13), GSE166488 (14), and GSE131411 (15) (The details of the datasets are shown in **Table 1**).

**Table 1:** Datasets and sample information.

GEO number	Species	Sample information	Microarray sequencing platform	Publication date (year)	Data source
GSE63311	Homo sapiens	Whole blood; sepsis (74) /Control (9)	Illumina Genome Analyzer IIx (Homo sapiens)	2014	Centenary Institute, Camperdown, Australia
GSE137340	Homo sapiens	Whole blood; sepsis (45) /Control (12)	Illumina HumanHT-12 V4.0 expression beadchip	2022	NATIONAL INSTITUTE OF BIOMEDICAL GENOMICS, Kalyani, India
GSE166488	Mus musculus	Liver; LPS (5)/Sham (3)	[Mouse430_2] Affymetrix Mouse Genome 430 2.0 Array	2021	Heidelberg University, Heidelberg, Germany
GSE184167	Mus musculus	Liver; CLP (3)/Sham (3)	Illumina NovaSeq 6000 (Mus musculus)	2021	Columbia University, New York, USA
GSE131411	Homo sapiens	Whole blood; septic shock (21)/cardiogenic shock (11)	Illumina Genome Analyzer IIx (Homo sapiens)	2020	SR-TIGET, Milano, Italy

To ensure consistency, two datasets, GSE137340 and GSE63311, were selected. The GSE137340 dataset included 22 cases collected 24 hours after sepsis diagnosis and 12 whole blood samples from healthy controls. The GSE63311 dataset included samples from four patients with SLI and nine healthy controls.

### 2.2 Identification of differentially expressed genes

GEO2R (https://www.ncbi.nlm.nih.gov/geo/geo2r) is an interactive online tool for screening DEGs between the treatment and control groups. All DEGs screened had a *p*–*value* of <0.05 and an absolute log2 fold-change (log2FC) > 1. Volcano plots were generated using GraphPad to visualize the DEGs identified in all four datasets. Venn diagrams were plotted using the online tool bioinformatics (bioinformatics.com.cn) (16).

### 2.3 DEGs function and pathway enrichment analysis

The DAVID database (https://www.david.ncifcrf.gov) contains biological data and analysis tools (17). In this study, we utilized the DAVID online bioinformatics database for GO functional analysis and KEGG analysis to identify the functions of DEGs. A *p*–*value* of <0.05 was designated as the threshold for significant enrichment.

### 2.4 Construction and analysis of PPI networks

The online database search tool STRING (https://www.string-db.org) enables access to protein structure and protein–protein association information in the genome (version 12.0) (18). We used STRING to construct a PPI network for the DEGs by designating a composite score greater than 0.4 as statistically significant. Cytoscape is a bioinformatics software package used used for biological network visualization and data integration (19). We imported the PPI data into Cytoscape (version v3.10.0) to map the PPI network and used the CytoHubba plugin (version 0.1) within the software to filter out the 11 core genes using Degree >= 6 as the criterion.

### 2.5 Correlation analysis of core DEGs with liver disease, infection, and immunity

The Comparative Toxicogenomics Database (CTD; https://www.ctdbase.org/) is a publicly available digital ecosystem that facilitates linkage between various chemicals, genes, diseases, and phenotypes (20). We used the CTD database to analyze the associations between the top 11 core DEGs identified and liver diseases, infections, and immune responses.

### 2.6 Immune infiltration analysis

We utilized R programming language and the CIBERSORT deconvolution algorithm to screen reliable samples with a *p*–value < 0.05 and assess the infiltration of 22 immune cell subsets in the serum of sepsis patients from the GSE137340 dataset. The “ggplot2” and “corrplot” packages in R were employed to visualize the infiltration levels of each immune cell subpopulation. Additionally, the “vioplot” package was used to generate violin plots illustrating changes in the infiltration of 22 immune cell types between sepsis patients and healthy controls.

Finally, Spearman correlation analysis was performed to calculate the relationships between potential biomarkers and immune cells. The visualization of these results was carried out using the “ggplot2” and “ggpubr” packages.

### 2.7 Construction of the murine sepsis model

The SLI model was established by CLP combined with intraperitoneal LPS injection. Male C57BL/6J mice (6-8 weeks old, 20-25 g) were housed in specific pathogen-free (SPF) facilities under a 12-hour light/dark cycle, with controlled temperature (25 ± 1°C), humidity (50 ± 5%), and ad libitum access to food and water. After a 7-day acclimatization period, the mice were randomly assigned to two groups.

#### 2.7.1 CLP treatment group

In the model group (n=8), mice were anesthetized by intraperitoneal injection of sodium pentobarbital (50 mg/kg). A 1.0-cm longitudinal incision was made along the midline of the lower abdomen. The cecum was gently exteriorized using blunt forceps, ligated along the midpoint of cecum with 4-0 silk thread, and punctured twice with a 21-gauge needle. Fecal contents were gently extruded through the puncture sites to ensure patency. The cecum was repositioned into the abdominal cavity, and the incision was sutured in layers. Sham-operated mice (n=8) underwent identical procedures except for cecal ligation and puncture. Postoperatively, 1 mL of sterile saline was administered subcutaneously in the neck region for fluid resuscitation (21).

#### 2.7.2 LPS treatment group

Mice in the model group (n=8) received an intraperitoneal injection of lipopolysaccharide (LPS; L2880, Sigma-Aldrich, St. Louis, MO, USA; 20 mg/kg), while mice in the control group (n=8) were administered an equivalent volume of phosphate-buffered saline (PBS) (22).

At approximately 8 hours post-modeling, mice in both groups exhibited characteristic sepsis symptoms, including fever, chills, piloerection, lethargy, and reduced locomotor activity. Twenty-four hours after modeling, all mice were anesthetized with sodium pentobarbital (50 mg/kg), and retro-orbital venous plexus blood sampling was performed. Subsequently, euthanasia was conducted via cervical dislocation, followed by liver tissue collection. A subset of liver samples was fixed in 4% paraformaldehyde, processed for hematoxylin and eosin (H&E) staining, and sectioned for histopathological evaluation. The remaining tissues were snap-frozen in liquid nitrogen and stored at −80°C for subsequent molecular analyses.

Serum samples were analyzed using a Hitachi automatic biochemical analyzer (Model 3100), with reagents provided by Mike Biotechnology Co., Ltd. The following parameters were measured: (1) Alanine aminotransferase (ALT): Determined using the alanine substrate method (CH0101201, Shandong, China). (2) Aspartate aminotransferase (AST): Determined using the aspartate substrate method (CH0101202, Shandong, China).

### 2.8 Quantitative real-time PCR validation of gene expression

Total RNA was extracted from liver tissue using TRIzol reagent (TIANGEN BIOTECH, Beijing, China), and reverse transcription was carried out using a reverse transcription kit from the same manufacturer. SYBR Green Mix (TIANGEN BIOTECH, Beijing, China) was utilized for real-time polymerase chain reaction. qRT–PCR was performed using a qTower3G real-time fluorescent quantitative PCR detection system (Analytik Jena AG, Germany). β-Actin expression was used as an internal control. Fold-change in relative expression levels was determined using the 2^−ΔΔCT^ method. All experiments were performed independently and repeated three times. The sequences of the primers are listed in **Supplementary Table S1**.

### 2.9 Statistical analysis

GraphPad Prism software (version 9.5.1; GraphPad Software, La Jolla, CA, USA) was utilized for the statistical analysis. All data are presented as the means ± standard deviations (SD). A Student’s t-test was used. All animal experiments were conducted with at least three replicates. A *p*–value of less than 0.05 was considered to indicate statistical significance.

## 3 Results

### 3.1 Identification and enrichment analysis of DEGs in patients with SLI

**Figure 1:**
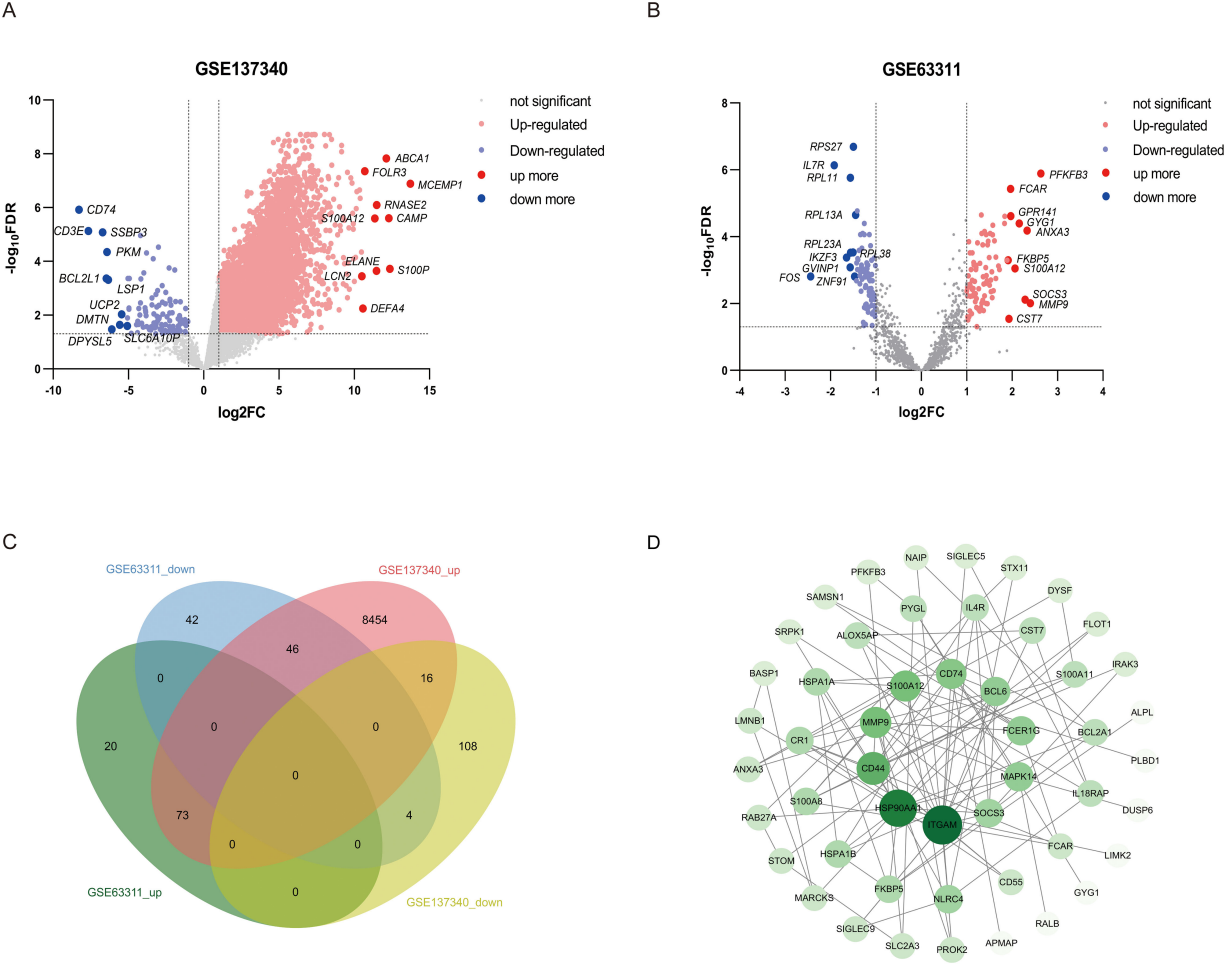
Visualization of human sepsis-associated liver injury datasets and screening of core differential genes. **(A, B)** The GSE63311 and GSE137340 were visualized using volcano plots. Upregulated genes are marked in red, downregulated genes are marked in blue, and gray indicates no change in gene expression levels. The top 10 upregulated and downregulated genes are labeled with gene symbols. **(C)** Venn analysis was used to compare the GSE63311 and GSE137340 datasets. **(D)** A PPI network was constructed using Cytoscape to visualize the intersection of the DEGs. Individual nodes in the PPI network were sorted based on their degree centrality, with darker colors and larger areas indicating higher centrality and greater importance.

The microarray results were visualized using volcano plots after normalization with GEO2R (**Figures 1A, B**). Statistical analyses were conducted with a *p*–value threshold of <0.05 and an absolute log2FC > 1. In the GSE63311 dataset, there were 93 upregulated and 92 downregulated genes, whereas the GSE137340 dataset had 10,291 upregulated and 143 downregulated genes. Venn analysis of the two datasets identified 77 intersecting DEGs, consisting of 73 upregulated and 4 downregulated genes (**Figure 1C**). GO enrichment analysis revealed that these 77 DEGs were primarily associated with functions such as the apoptotic process and its negative regulation, positive regulation of NF-κB transcription factor activity, positive regulation of inflammatory response and positive regulation of protein phosphorylation, response to bacteria (**Supplementary Figure S1A**). KEGG pathway enrichment analysis indicated that these DEGs were primarily involved in pathways such as IL-17 signaling, antigen processing and presentation, estrogen signaling, lipid and atherosclerosis, and hematological disorders (**Supplementary Figure S1B**). Cellular components (CC) and molecular function (MF) are shown in the supplementary diagrams (**Supplementary Figures S1C, D**). A PPI network was constructed using String and Cytoscape to visualize the relationships among these 77 DEGs. Based on a degree >= 6 criterion, 11 core genes were identified (*ITGAM*, *HSP90AA1*, *CD44*, *MMP9*, *CD74*, *MAPK14*, *BCL6*, *FCER1G*, *NLRC4*, S100A12 and *SOCS3*. **Figure 1D**). The correlation of these 11 DEGs with liver disease, infection, sepsis, and immunity was assessed using the CTD database, showing significant correlations between liver disease and sepsis, as well as these core genes and the immune response (**Supplementary Table S2**).

### 3.2 Immune cell infiltration analysis

**Figure 2:**
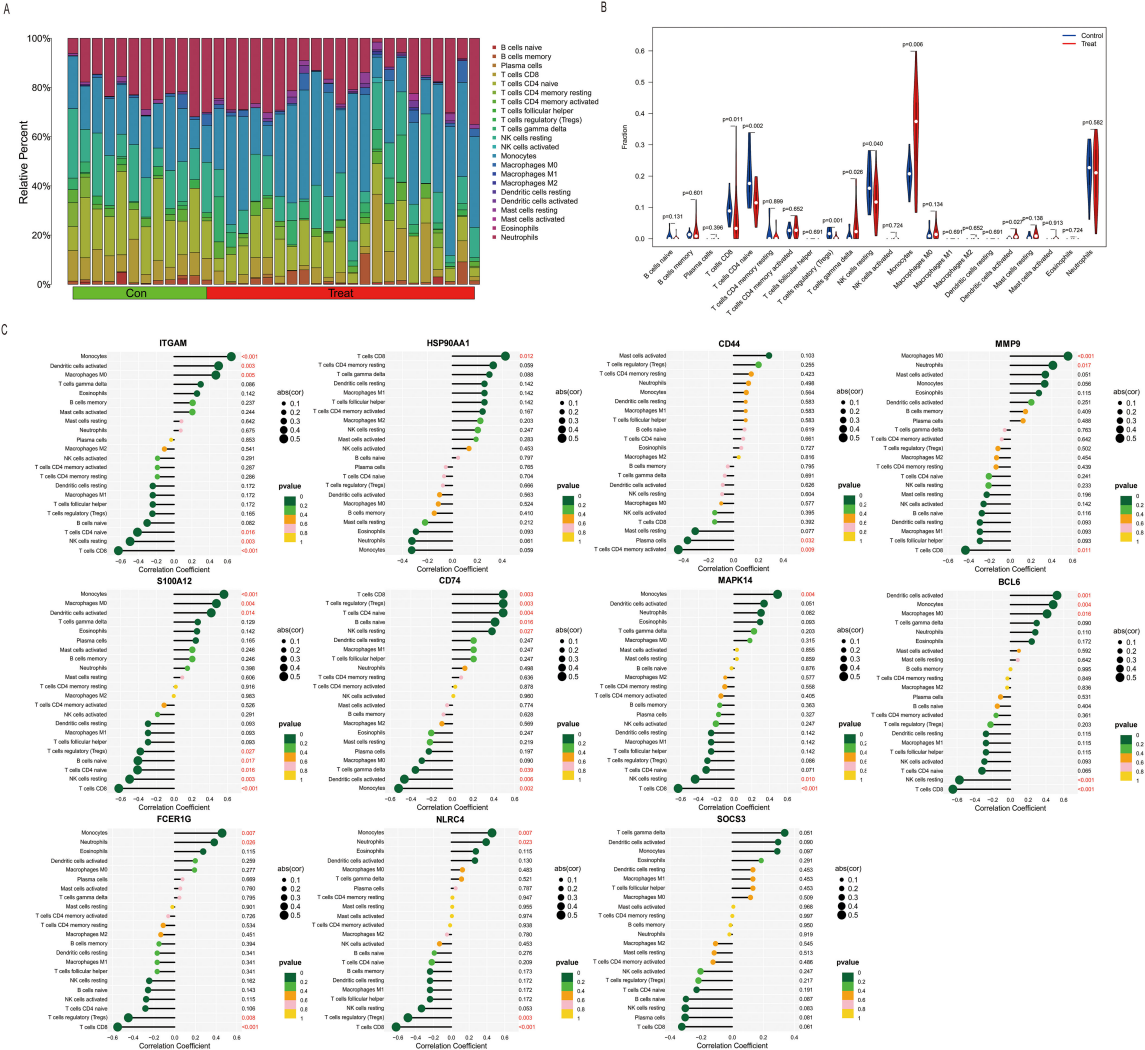
Correlation of sepsis and core DEGs with immune cell infiltration. **(A)** Histogram showing the proportions of infiltration for 22 types of immune cells. **(B)** Differences in immune infiltration between normal and septic patient sera (blue represents the normal control group, and red represents the sepsis group. A *p*–*value* of <0.05). **(C)** Immune infiltration levels of the 22 immune cell types were obtained using the single-sample gene set enrichment analysis (ssGSEA) algorithm. Lollipop plots analyzed the correlation between the expression levels of *ITGAM*, *HSP90AA1*, *CD44*, *MMP9*, *S100A12*, *CD74*, *MAPK14*, *BCL6*, *FCER1G*, *NLRC4*, and *SOCS3* and the infiltration of 22 immune cell types.

Data from the GSE137340 dataset were analyzed using the CIBERSORT Transposed Convolution method with a *p*–value cutoff of < 0.05, yielding the relative proportions of 22 immune cell infiltrations in the serum samples of 34 patients from both the sepsis and control groups (**Figure 2A**). The results of the Wilcoxon test indicated that, compared to the control group, the infiltration rates of γδ T cells, monocytes, and activated dendritic cells were significantly increased in patients with sepsis, while the infiltration rates of CD8 T cells, naïve CD4 T cells, regulatory T cells (Tregs), and resting NK cells were significantly decreased (**Figure 2B**). Subsequently, we analyzed the correlation between 11 core genes and immune cells. The results revealed that, with the exception of *SOCS3*, all other genes were significantly correlated with immune cells. Specifically, *ITGAM, S100A12, MAPK14, BCL6, FCER1G*, and *NLRC4* showed significant positive correlations with monocytes. Additionally, *ITGAM, S100A12* and *BCL6* were positively correlated with M0 macrophages and activated dendritic cells. Conversely, *ITGAM, MMP9, S100A12, MAPK14, BCL6, FCER1G* and *NLRC4* demonstrated significant negative correlations with CD8 T cells. Both *ITGAM* and *S100A12* were significantly negatively correlated with naïve CD4 T cells. Furthermore, *ITGAM, S100A12, MAPK14* and *BCL6* exhibited significant negative correlations with resting NK cells (**Figure 2C**). The correlations between core genes and immune cell infiltration reveal a complex immune landscape in sepsis-related liver injury. Positive correlations with monocytes and activated dendritic cells (e.g., *ITGAM, S100A12, MAPK14*) suggest roles in innate immunity modulation, while negative correlations with CD8 T cells, naïve CD4 T cells, and resting NK cells indicate immune dysregulation and suppression of adaptive immunity. These core genes may be key modulators in the immune response, influencing both inflammation and immune regulation in liver injury during sepsis.

### 3.3 Identification and enrichment of DEGs in mice with SLI

#### 3.3.1 Identification and functional enrichment of DEGs in the CLP model

**Figure 3:**
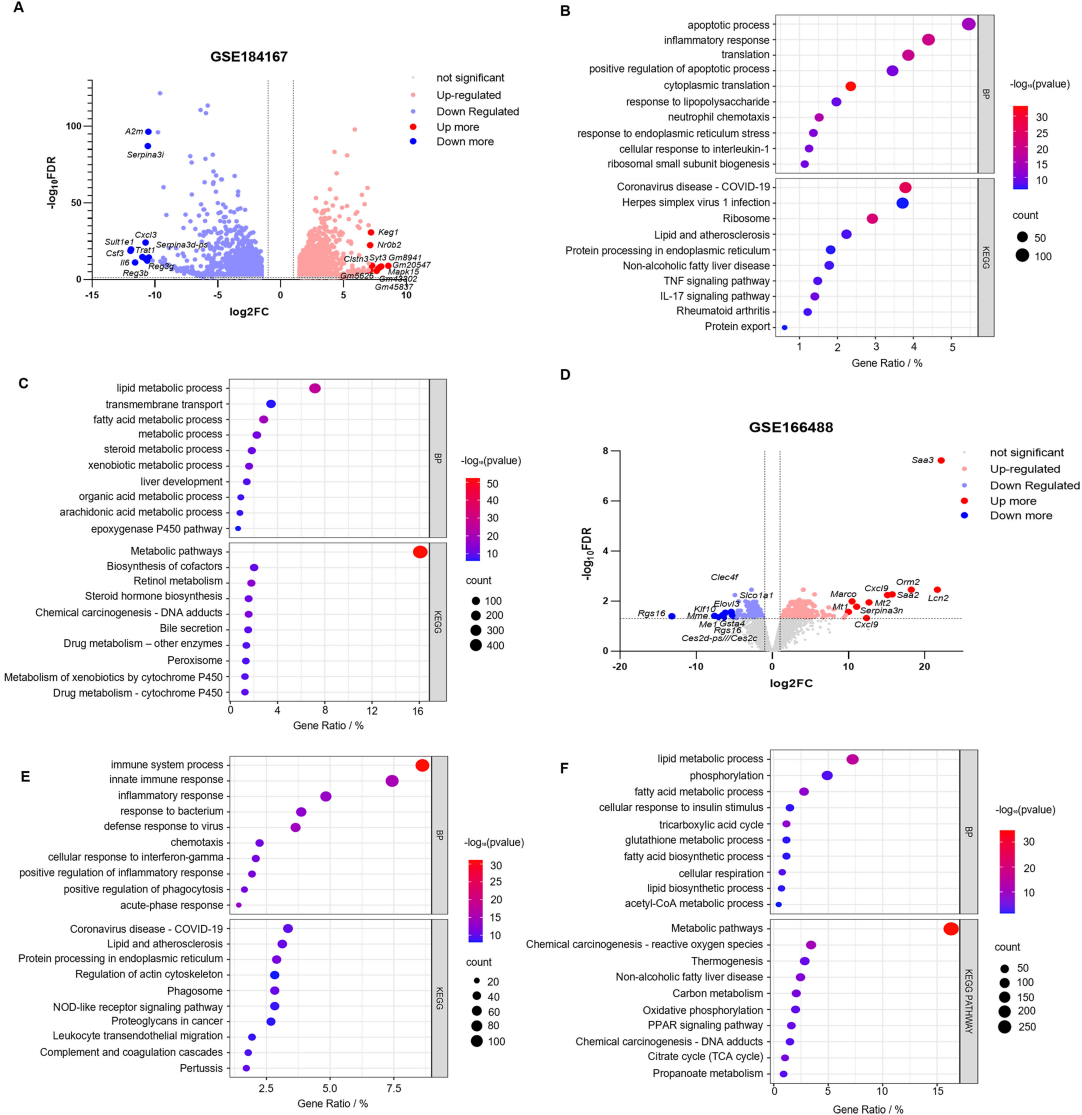
Identification and correlation analysis of DEGs in SLI in the mouse CLP and LPS models. **(A, B)** Volcano plots of the GSE184167 and GSE166488 datasets. Gene symbols are used to label the top 10 upregulated and downregulated genes, with upregulated genes indicated in red, downregulated genes in blue, and no change in gene expression level represented by gray. **(C, D)** The top 10 upregulated genes identified through BP and KEGG pathway analysis in the GSE184167 and GSE166488 datasets. **(E, F)** The top 10 downregulated genes identified through BP and KEGG pathway analysis in the GSE184167 and GSE166488 datasets.

Limited availability of human tissue samples and ethical considerations necessitate the widespread use of mouse models for studying human disease pathogenesis. To elucidate the mechanisms underlying SLI, we employed the CLP-induced SLI model, widely regarded as the “gold standard” for sepsis research (23), to assess its accuracy in replicating human pathophysiological changes. The GSE184167 dataset was retrieved from the GEO database and filtered using thresholds of |log2FC| > 1 and p < 0.05, identifying 2737 upregulated and 2722 downregulated genes (**Figure 3A**). Integrated GO and KEGG enrichment analyses consistently revealed that upregulated DEGs were significantly enriched in inflammatory responses, apoptotic processes, immune activation pathways (e.g., IL-17 and TNF signaling), and endoplasmic reticulum stress pathways (**Figure 3C**), while downregulated DEGs predominantly clustered within critical hepatic metabolic functions including lipid/fatty acid/steroid metabolism and bile secretion (**Figure 3D**); CC and MF analyses for this dataset are provided in **Supplementary Figures S2A** and **S2B**. Functional assessment demonstrated that the marked upregulation of inflammation-, apoptosis-, and immunity-associated genes (including key mediators of IL-17/TNF pathways) in CLP-modeled mice closely aligns with core pathological features of human SLI—exaggerated inflammatory responses and tissue damage (24, 25). Concurrently, downregulation of genes governing essential metabolic pathways (e.g., lipid metabolism, bile secretion) precisely mirrors the characteristic hepatocyte functional suppression observed in human sepsis (26). Collectively, these molecular-level findings indicate that the CLP model faithfully recapitulates key pathophysiological mechanisms of human SLI, particularly the core features of inflammation/immune activation coupled with metabolic dysfunction, thereby validating its utility as an effective tool for investigating human SLI pathogenesis.

#### 3.3.2 Identification and functional enrichment of DEGs using the LPS intraperitoneal injection model

While the CLP model construction method closely resembles the pathophysiological process of human sepsis, achieving standardization can be challenging, and the success of the surgery is highly dependent on the user’s technical ability (27). In contrast, the intraperitoneal injection of LPS is widely used to generate sepsis models. This method is more stable and triggers a more significant inflammatory response (28). Therefore, we downloaded the GSE166488 dataset from the GEO database and filtered the data using an absolute log2FC > 1 and a *p*–value of <0.05. Upregulated and downregulated genes numbered 1,831 and 1,979, respectively (**Figure 3B**). GO enrichment analysis revealed that the upregulated genes were primarily associated with the immune response, defense response to viruses, acute response, inflammatory response, and the positive regulation of it, cellular response to interferon, positive regulation of phagocytosis, and chemotaxis (**Figure 3E**). The downregulated genes were primarily focused on metabolic processes, including lipid and fatty acid metabolism and biosynthesis, phosphorylation, glutathione metabolism, and acetyl coenzyme metabolism (**Figure 3F**). KEGG pathway enrichment analysis revealed that the upregulated DEGs were primarily involved in protein processing in the endoplasmic reticulum, complement coagulation cascade reaction, nod-like receptor signaling pathway, and leukocyte migration (**Figure 3E**). Downregulated DEGs were associated with metabolic pathways, including carbon metabolism, the citric acid cycle, PPAR signaling pathway, oxidative phosphorylation, and propionic acid metabolism (**Figure 3F**). The CC and MF of the GSE166488 are shown in the supplementary diagram (**Supplementary Figures S2C, D**). The LPS model effectively recapitulated core inflammatory pathology of human SLI through activation of inflammatory/immune pathways and partial metabolic alterations. Compared to CLP, LPS demonstrated stronger enrichment of inflammation-associated DEGs, providing superior capability in modeling disease-specific inflammatory responses.

#### 3.3.3 Both mouse models partially reproduced the mechanism of liver injury in human sepsis

**Figure 4:**
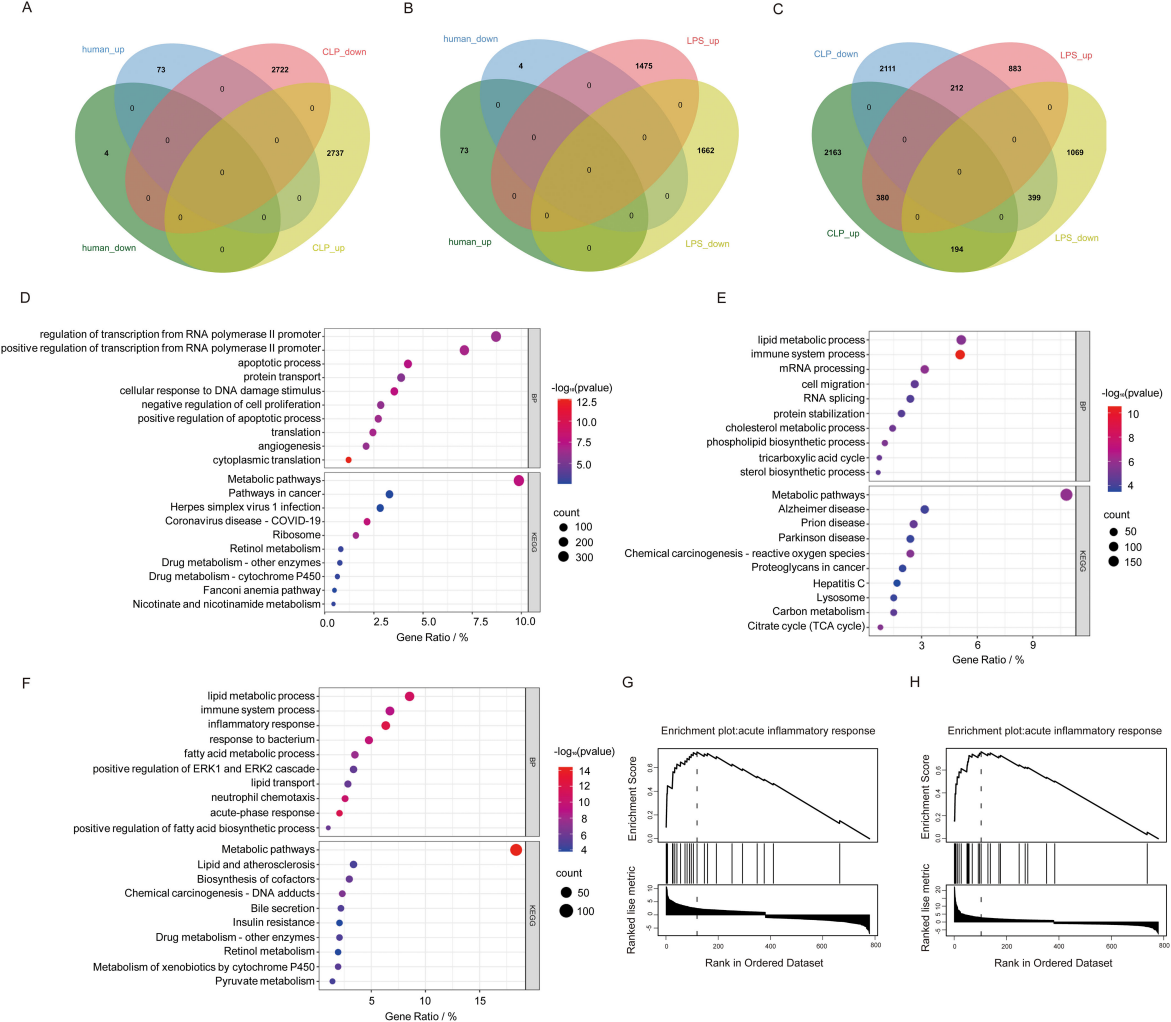
Comparison of human sepsis DEGs between DEGs from two mouse sepsis liver injury models. **(A)** Venn analysis of DEGs common to human sepsis and the CLP mouse model of liver injury. **(B)** Venn analysis of DEGs common to human sepsis and the LPS mouse model of sepsis liver injury. **(C)** Venn analysis of DEGs shared between human sepsis and the CLP mouse model of liver injury. **(D)** The top 10 DEGs specific to the CLP model. **(E)** The top 10 DEGs specific to the LPS model. **(F)** The shared genes between the two models based on BP and KEGG analyses. **(G, H)** GSEA enrichment analysis of the intersecting genes associated with inflammatory response mechanisms in the CLP and LPS models.

Integrated analysis reveals fundamental mechanistic distinctions between mouse SLI models and human samples despite lacking overlapping DEGs. Venn diagram analysis identified 7,794 model-specific DEGs (CLP: 4,274; LPS: 1,952) and 779 shared genes (**Figures 4A–C**). Shared DEGs mediate core inflammatory/metabolic processes (e.g., acute-phase response, neutrophil chemotaxis; **Figure 4D**), whereas CLP-specific DEGs (e.g., *NLRP3, CASP1, VEGFA*) enrich apoptosis/angiogenesis pathways (**Figure 4E**), recapitulating polymicrobial sepsis complexity via inflammasome-mediated pyroptosis and vascular remodeling (29). Conversely, LPS-specific DEGs (e.g., *CXCL1, CXCL9, CSRP3*) primarily regulate TLR4/NF-κB-driven cytokine storms (**Figure 4F**), enabling rapid innate immune activation ideal for acute intervention studies (30). GSEA confirmed acute inflammation as a shared process with model-specific signatures (**Figures 4G, H**): *CXCL1* dominates neutrophil recruitment in LPS (31), while VEGFA governs CLP-specific vascular repair (32). This functional dichotomy defines complementary model value—LPS uniquely models cytokine-driven acute injury for therapeutic screening (30), while CLP authentically replicates subacute repair processes through inflammasome/angiogenesis axes (29, 32).

### 3.4 *SOCS3* was significantly increased in both mouse models of SLI

**Figure 5:**
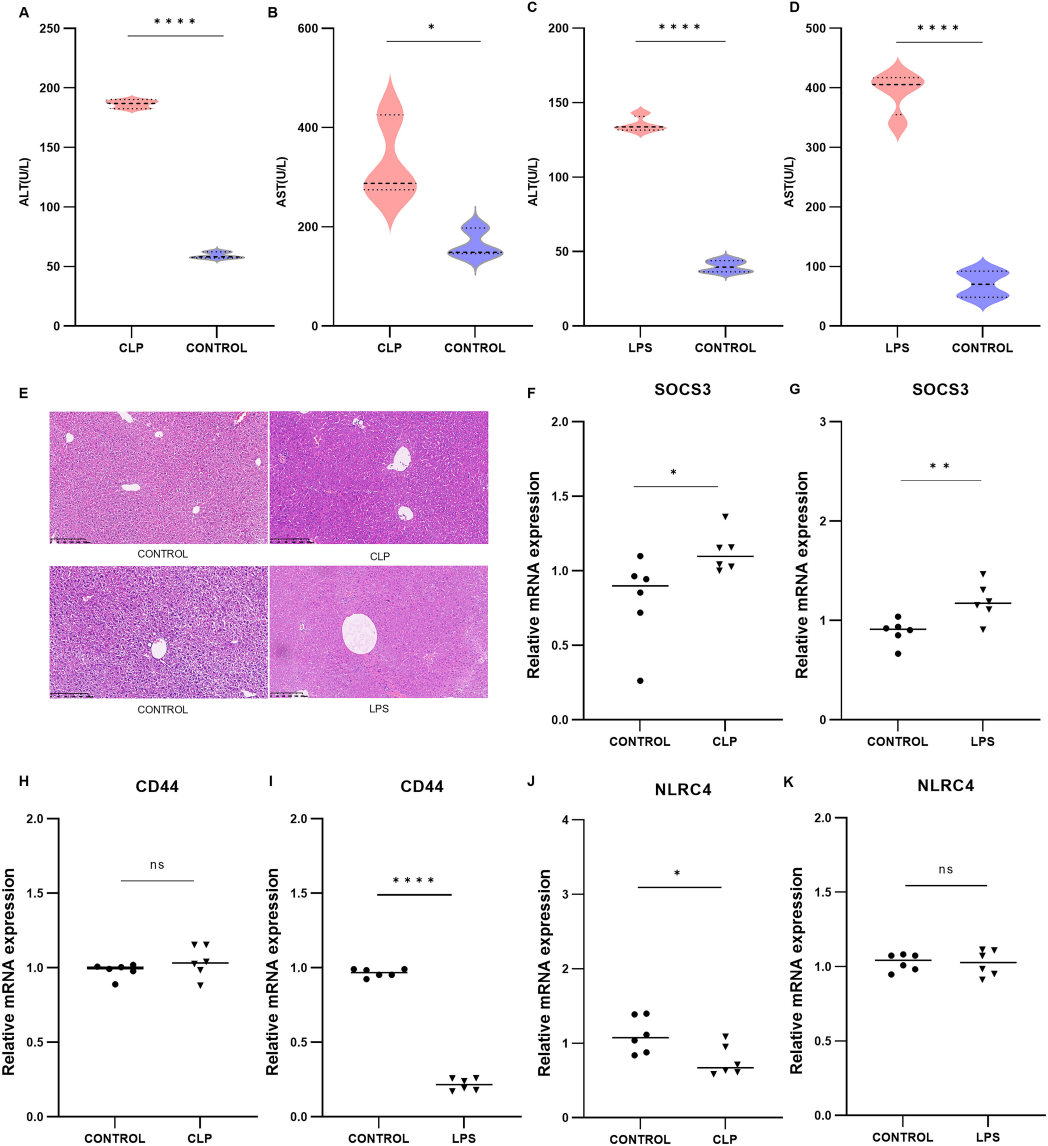
Validation of mouse CLP models (n=3) and LPS models (n=3) and the expression of core DEGs. **(A, B)** Serum ALT and AST levels in the CLP group. **(C, D)** Serum ALT and AST levels in the LPS group. **(E)** H&E-stained image of livers from the CLP and LPS groups. **(F–K)** Relative mRNA expression levels of *SOCS3*, *ITGAM*, *MMP9, BCL6, FCER1G* in the CLP and LPS groups. *p < 0.05, **p < 0.01, ****p < 0.0001.

To validate the core biomarkers associated with SLI, SLI models were established using CLP and intraperitoneal injection of LPS. The successful establishment of these models was confirmed by measuring serum ALT and AST levels and conducting a histological examination of the liver using H&E staining. The results demonstrated that there was a significant increase in serum ALT and AST levels in both models compared with the control group (**Figures 5A–D**, A: *p* < 0.0001; B: *p* = 0.0319; C: *p* < 0.0001; D: *p* < 0.0001). Histopathological analysis of the liver revealed disrupted liver structure, hepatocyte swelling, necrosis, and inflammatory cell infiltration in the experimental group compared with the control group (**Figure 5E**). Eleven core genes with a connectivity degree of 6 or higher were identified (*ITGAM, HSP90AA1, CD44, MMP9, CD74, MAPK14, BCL6, FCER1G, NLRC4, SOCS3, S100A12*), and their expression was validated in two animal models using PCR, with the exception of S100A12, which is not expressed in mice (33).The results indicated that, among these 10 core genes, only *SOCS3* was significantly upregulated in both SLI models (**Figures 5F, G**, F: *p* = 0.0377; G: *p* = 0.0080). *CD44* was differentially expressed only in the LPS group (**Figures 5H, I**, H: *p* = 0.2432; I: *p* < 0.0001), whereas *NLRC4* was significantly expressed only in the CLP group (**Figures 5J, K**, J: *p* = 0.0245; K: *p* = 0.9164). The other genes exhibited no significant differences (**Supplementary Figure S6**). *SOCS3* may serve as a potential biomarker for SLI.

### 3.5 The expression of *SOCS3* in patients with sepsis changed with time

**Figure 6:**
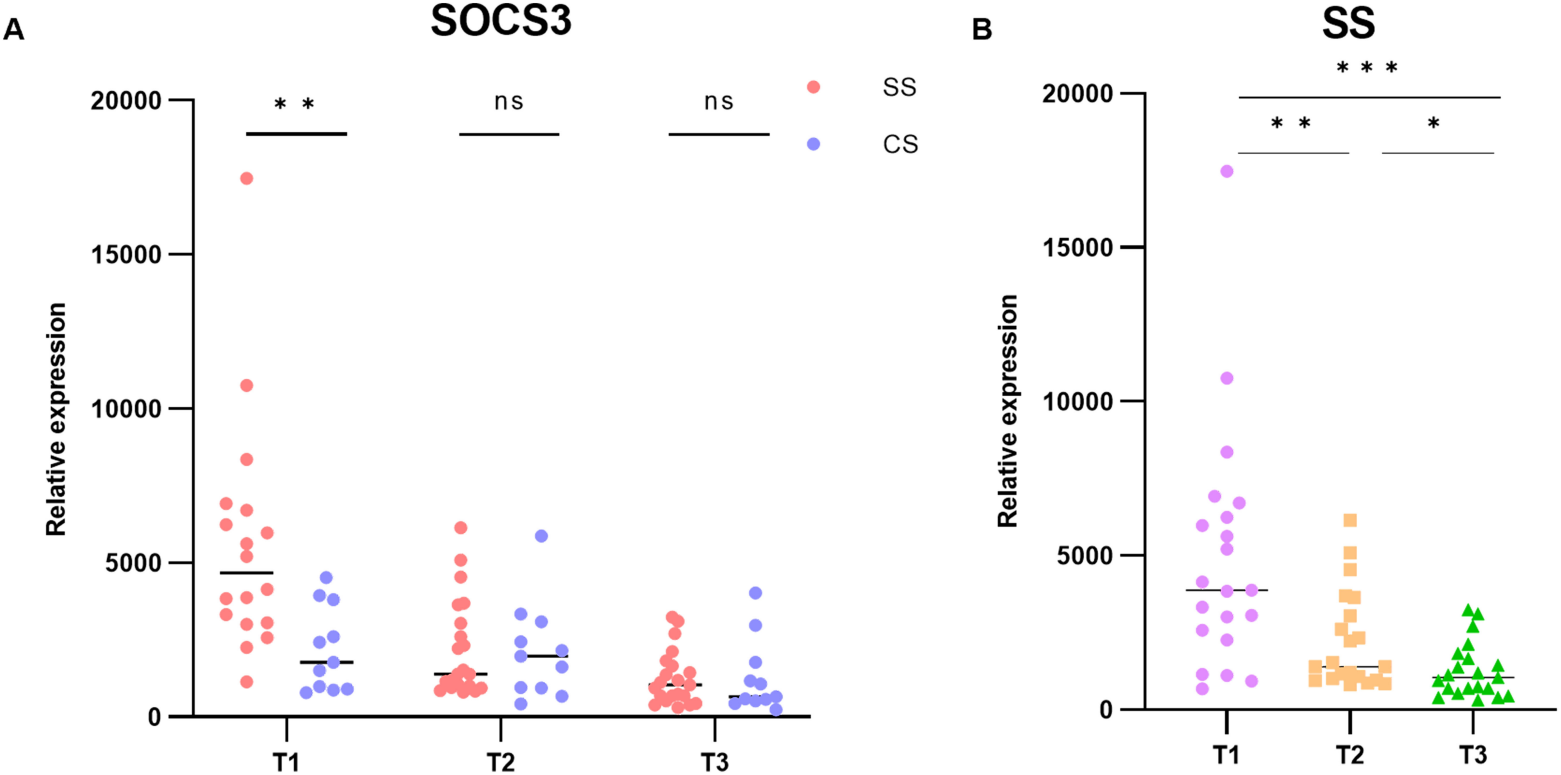
Temporal characterization of *SOCS3* expression in human SLI. **(A)** Expression of *SOCS3* in T1, T2, and T3 time points. **(B)** Changes in *SOCS3* expression in patients with SS at T1, T2, and T3 time points. *p < 0.05, **p < 0.01, ***p < 0.001.

To examine the temporal characteristics of *SOCS3* expression in patients with sepsis, we analyzed the GSE131411 dataset obtained from the GEO database. Changes in *SOCS3* expression were determined in patients with septic shock (SS) and cardiogenic shock (CS) at the following three time points: within 16 h of ICU admission (T1), 48 h after study enrollment (T2), and on day 7 after ICU admission or before discharge from the ICU (T3). The results indicated that *SOCS3* was significantly upregulated in patients with SS compared with those with CS, with significant differences observed at T1 (**Figure 6A**, SS vs. CS: T1: *p* = 0.0075; T2: *p* = 0.9079; T3: *p* = 0.8465). Furthermore, we evaluated the temporal changes in *SOCS3* expression within the septic shock group at T1, T2, and T3. *SOCS3* expression exhibited the most significant increase during the early stage of sepsis, with statistically significant differences compared with the T2 and T3 periods (**Figure 6B**, T1 vs. T2: *p* = 0.0052; T1 vs. T3: *p* = 0.0002; T2 vs. T3: *p* = 0.0222).

## 4 Discussion

The pathogenesis and treatment of sepsis remain challenging. As a main target of sepsis, the liver plays an important role in regulating the inflammatory response and maintaining immune homeostasis, and SLI is considered a prognostic indicator of sepsis (34–36). Currently, sepsis has been extensively studied, but the specific biomarkers for diagnosing and treating liver injury in sepsis are still not well understood. At present, the clinical diagnosis of SLI mainly relies on clinical manifestations and related biochemical indicators [such as C-reactive protein, procalcitonin (PCT), tumor necrosis factor α (TNF-α), and interleukin-6 (IL-6)] for comprehensive assessment (37–40). However, these methods are not highly sensitive, and the therapeutic effects are not ideal. Therefore, we explored specific biomarkers for septic liver damage and their correlation with immune cell infiltration through comprehensive and objective biological information, with a view to providing new insights into the detection and diagnosis of sepsis.

The pathogenesis of SLI is driven by interconnected immune dysregulation cascades wherein innate immune hyperactivation manifests as monocyte-derived macrophage adherence to hepatic sinusoids via *ITGAM/CD44*-mediated mechanisms (41), promoting TLR4/NF-κB-dependent M1 polarization that amplifies cytokine storms and mitochondrial dysfunction to directly induce hepatocyte death (42, 43). Concurrently, *MMP9*-activated γδ T-cell/neutrophil axis triggers neutrophil infiltration and NETosis formation (44), exacerbating sinusoidal obstruction and hypoxic injury. Adaptive immunity fails through *CD74/FCER1G*-regulated dendritic cell and T-cell hyperactivation (45), which disrupts antigen presentation to induce CD8^+^T-cell exhaustion and Treg suppression, ultimately crippling immune surveillance. Compounding this pathology, insufficient IL-10 production and aberrant *SOCS3* signaling impede anti-inflammatory M2 macrophage conversion (46), perpetuating tissue damage. These processes collectively establish a self-amplifying inflammation-damage-immunoparalysis cycle (38). Our experimental models provide complementary validation: the LPS model optimally recapitulates acute TLR4/NF-κB-dominated innate hyperinflammation, while the CLP model more faithfully mirrors progressive human SLI dynamics including T-cell exhaustion and repair mechanisms. Crucially, ten hub genes (*ITGAM, CD44, MMP9, CD74, FCER1G, MAPK14, BCL6, NLRC4, S100A12*) mechanistically bridge specific immune cell subsets, confirming their roles as central orchestrators of SLI’s immune landscape.

To further elucidate the pathogenesis of SLI, we analyzed mouse sepsis liver injury datasets constructed using CLP and LPS models from the GEO database. DEGs and their underlying molecular mechanisms were compared between these datasets and human datasets. Venn analysis revealed no overlapping DEGs between animal models and human serum samples. However, functional predictions showed that both mouse models contained DEGs associated with inflammatory responses, bacterial responses, and immune processes, which are also involved in the development of human sepsis-induced liver injury. This suggests a common mechanism by which these models replicate SLI, as both can reproduce certain phenotypes of human SLI through the processes described above. In addition, previous studies have shown distinct characteristics between the two models, In the LPS model, direct intraperitoneal injection of endotoxin induces a rapid and intense inflammatory response, whereas in the CLP model, direct exposure to the mouse’s own intestinal microbiota via the abdominal cavity triggers sepsis and associated liver injury (27). The latter more closely resembles the complex and diverse pathophysiological processes of human sepsis (47, 48). Our study further demonstrated that, compared to the LPS model, the DEGs in the CLP model were involved in a broader range of biological processes and pathways in the progression of sepsis, including the regulation of protein expression and transport, as well as the initiation of cell death (49). Based on these findings, we concluded that the CLP model better reflects the pathophysiological characteristics of human sepsis. Finally, we validated the expression of core hub genes (excluding *S100A12*) in the two mouse models using qRT-PCR, identifying *SOCS3* as a potential key biomarker of sepsis-induced liver injury. Additionally, we analyzed temporal changes in *SOCS3* expression during sepsis. By comparing transcriptomic changes in whole blood from patients with septic shock and cardiogenic shock, we observed that *SOCS3* expression was significantly upregulated in the early stages of sepsis. This further supports the hypothesis that *SOCS3* may serve as a biomarker of liver injury in human sepsis.

*SOCS3* is a member of the *SOCS* family of proteins and acts as a key negative regulator in various biological processes (50). Studies have shown that *SOCS3* expression exerts a protective effect on endothelial cells during sepsis, and its deficiency leads to endothelial dysfunction, thereby exacerbating tissue injury (51). Additionally, *SOCS3* mitigates inflammatory damage by promoting macrophage polarization to the M2 phenotype, maintaining vascular homeostasis, and improving survival rates in sepsis patients (52). Moreover, *SOCS3* plays a critical role in liver repair after injury by inhibiting IL-6-induced JAK/STAT signaling, which suppresses hepatocyte proliferation (53). Our results demonstrated that SLI is closely associated with *SOCS3* and overall immune function. However, immune correlation analysis revealed no significant association between *SOCS3* and the 22 types of immune cells analyzed, which contradicts findings from previous studies (54). This discrepancy may be attributed to factors such as heterogeneity in disease types and states, limited sample size, and variations in computational algorithms. Further research is needed to clarify these differences. In conclusion, *SOCS3* likely plays a critical regulatory role in the development and progression of SLI through various mechanisms. These findings offer valuable insights and potential directions for future therapeutic strategies.This mechanism prevents excessive regeneration and reduces the risk of malignant transformation. Taken together, *SOCS3* appears to play a pivotal role in regulating the pathogenesis of SLI through various mechanisms, providing new avenues for future therapeutic strategies. Notably, PS Grutkoski et al. first demonstrated that *SOCS3* is upregulated and exhibits time-dependent expression in a mouse model of sepsis (55). Consistent with this, our study confirmed the time-dependent nature of *SOCS3* expression and its association with liver injury, offering novel insights into the diagnosis and treatment of SLI.

In summary, this study highlights that the CLP model is more representative for studying human septicemic liver injury. Through bioinformatics analysis, we identified and validated several hub genes and, for the first time, proposed that *SOCS3* may serve as a biomarker for septicemia-induced liver injury. While the findings provide valuable insights, they require further validation in larger studies. This study has several limitations. Previous research has demonstrated genetic overlap and functional similarities between mouse sepsis models and LPS-induced transcriptional responses in human cells (56), suggesting that the transcriptional responses in mouse sepsis models mirror those observed in humans. However, in this study, no overlapping genes were identified between the human and mouse sepsis datasets, likely due to the heterogeneity of human tissue samples. Previous studies utilized human blood mononuclear cells and mouse peritoneal macrophages, while our analysis focused on whole human blood and mouse liver tissue, leading to differing results.Additionally, the datasets used in this study were obtained from public databases and were not assessed for data quality. Variability in datasets, platforms, and statistical methods may have influenced the reliability of the results. Furthermore, differences in model construction methods, sample size, age, race, and disease status may have reduced the overall integrity of the study. Despite these limitations, this study has identified potential biomarkers of SLI, offering valuable directions for future research into the pathogenesis and treatment of SLI.

## Data Availability

The datasets presented in this study can be found in online repositories. The names of the repository/repositories and accession number(s) can be found in the article/**Supplementary Material**.
