# Supplementary material for: Transcriptional responses in different mouse models of septic liver injury differ from those in patients with septic liver injury
Source: Front Immunol. 2025 Jul 30;16:1556392. doi: 10.3389/fimmu.2025.1556392 (PMC12343667; doi:10.3389/fimmu.2025.1556392)
Supplement: Supplementary file 1 [file DataSheet1.pdf]

Table S1. Primer sequences used for quantitative RT–PCR

Gene	Forward primer sepuence (5′ → 3′)	Rorward primer sepuence (5′ → 3′)
ITGAM	GGGAGGACAAAAACTGCCTCA	ACAACTAGGATCTTCGCAGCAT
HSP90AA1	GACGCTCTGGATAAAATCCGTT	TGGGAATGAGATTGATGTGCAG
CD44	AGAAAAATGGCCGCTACAGTATC	TGCATGTTTCAAAACCCTTGC
MMP9	CTGGACAGCCAGACACTAAAG	CTCGCGGCAAGTCTTCAGAG
CD74	AGTGCGACGAGAACGGTAAC	CGTTGGGGAACACACACCA
MAPK14	TGACCCTTATGACCAGTCCTTT	GTCAGGCTCTTCCACTCATCTAT
BCL6	TAGAGCCCATAAGACAGTGCT	CACCGCCATGATATTGCCTTC
FCER1G	ATCTCAGCCGTGATCTTGTTCT	ACCATACAAAAACAGGACAGCAT
NLRC4	GAAACACTGTACGATCAGCTCC	CATGTTCTTGAAGCGATGGTTTT
SOCS3	AAGACCTTCAGCTCCAAAAG	GAGCATCATACTGATCCAGG
β-Actin	GTCGTACCACAGGCATTGTGATGG	GCAATGCCTGGGTACATGGTGG

Table S2. Association of the eleven hub genes with infection and liver diseases (from the CTD database).

Gene	Disease name	Disease ID	Inference score	Inference count
ITGAM	Chemical and Drug Induced Liver Injury	MESH:D056486	336.82	1388
	Liver Diseases	MESH:D008107	148.7	117
	Liver Failure, Acute	MESH:D017114	71.54	208
	Liver Failure	MESH:D017093	47.62	33
	Bacterial Infections	MESH:D001424	16.72	6
	Infections	MESH:D007239	9.54	4
	Bacteremia	MESH:D016470	3.49	1
	Sepsis	MESH:D018805	45.8	44
	Immune System Diseases	MESH:D007154	76.1	24
	IMMUNE SUPPRESSION	OMIM:146850	7.85	3
HSP90AA1	Chemical and Drug Induced Liver Injury	MESH:D056486	389.27	1517
	Liver Diseases	MESH:D008107	143.64	116
	Liver Failure, Acute	MESH:D017114	80.32	217
	Liver Failure	MESH:D017093	58.05	41
	Infections	MESH:D007239	22.45	8
	Bacterial Infections	MESH:D001424	13.08	9
	Bacteremia	MESH:D016470	2.02	2
	Sepsis	MESH:D018805	40.74	31
	Immune System Diseases	MESH:D007154	126.22	39
	IMMUNE SUPPRESSION	OMIM:146850	6.86	2
CD44	Chemical and Drug Induced Liver Injury	MESH:D056486	405.3	1540
	Liver Diseases	MESH:D008107	167.22	121
	Liver Failure, Acute	MESH:D017114	78.45	211
	Liver Failure	MESH:D017093	56.84	41
	Bacterial Infections	MESH:D001424	39.8	14

	Infections	MESH:D007239	25.63	13
	Bacteremia	MESH:D016470	7.61	7
	Sepsis	MESH:D018805	57.64	52
	Immune System Diseases	MESH:D007154	68.21	27
	IMMUNE SUPPRESSION	OMIM:146850	11.42	2
MMP9	Chemical and Drug Induced Liver Injury	MESH:D056486	737.58	1835
	Liver Diseases	MESH:D008107	208.51	153
	Liver Failure, Acute	MESH:D017114	124.09	246
	Liver Failure	MESH:D017093	68.95	57
	Bacterial Infections	MESH:D001424	16.72	6
	Infections	MESH:D007239	9.54	4
	Bacteremia	MESH:D016470	6.99	4
	Sepsis	MESH:D018805	67.94	59
	Immune System Diseases	MESH:D007154	87.62	38
	IMMUNE SUPPRESSION	OMIM:146850	8.5	4
S100A12	Chemical and Drug Induced Liver Injury	MESH:D056486	51	613
	Liver Diseases	MESH:D008107	26.85	34
	Liver Failure, Acute	MESH:D017114	13.31	153
	Liver Failure	MESH:D017093	11.1	13
	Infections	MESH:D007239	7.61	2
	Bacterial Infections	MESH:D001424	6.96	2
	Bacteremia	MESH:D016470	3.49	1
	Sepsis	MESH:D018805	6.21	17
	Immune System Diseases	MESH:D007154	29.45	10
CD74	Liver Failure, Acute	MESH:D017114	69.2	200
	Chemical and Drug Induced Liver Injury	MESH:D056486	302.62	1376
	Liver Diseases	MESH:D008107	118.91	98
	Liver Failure	MESH:D017093	49.33	34
	Infections	MESH:D007239	26.11	9
	Bacterial Infections	MESH:D001424	22.88	11
	Bacteremia	MESH:D016470	5.48	6
	Sepsis	MESH:D018805	32.9	29
	Immune System Diseases	MESH:D007154	83.77	30
	IMMUNE SUPPRESSION	OMIM:146850	7.59	3
MAPK14	Chemical and Drug Induced Liver Injury	MESH:D056486	496.23	1742
	Liver Diseases	MESH:D008107	215.32	150
	Liver Failure, Acute	MESH:D017114	132.59	236
	Liver Failure	MESH:D017093	68.23	47
	Bacterial Infections	MESH:D001424	40.37	11
	Infections	MESH:D007239	20.25	9
	Bacteremia	MESH:D016470	4.46	3
	Sepsis	MESH:D018805	49.58	53
	Immune System Diseases	MESH:D007154	82.45	27
	IMMUNE SUPPRESSION	OMIM:146850	15.57	4
BCL6	Chemical and Drug Induced Liver Injury	MESH:D056486	295.5	1412
	Liver Diseases	MESH:D008107	132.67	102

	Liver Failure, Acute	MESH:D017114	74	200
	Liver Failure	MESH:D017093	54.13	37
	Infections	MESH:D007239	26.35	7
	Bacterial Infections	MESH:D001424	15.91	9
	Sepsis	MESH:D018805	33.26	42
	Immune System Diseases	MESH:D007154	84.4	31
	IMMUNE SUPPRESSION	OMIM:146850	7.66	2
FCER1G	Chemical and Drug Induced Liver Injury	MESH:D056486	189.98	1275
	Liver Diseases	MESH:D008107	85.4	83
	Liver Failure, Acute	MESH:D017114	55.97	199
	Liver Failure	MESH:D017093	34.76	33
	Bacterial Infections	MESH:D001424	15.13	5
	Infections	MESH:D007239	9.12	2
	Sepsis	MESH:D018805	19.84	25
	Immune System Diseases	MESH:D007154	66.83	21
	IMMUNE SUPPRESSION	OMIM:146850	4.35	1
NLRC4	Chemical and Drug Induced Liver Injury	MESH:D056486	84.1	357
	Liver Diseases	MESH:D008107	39.62	22
	Liver Failure, Acute	MESH:D017114	10.5	15
	Liver Failure	MESH:D017093	2.43	1
	Bacterial Infections	MESH:D001424	13.66	4
	Infections	MESH:D007239	6.28	3
	Sepsis	MESH:D018805	4.92	17
	Immune System Diseases	MESH:D007154	52.21	20
	IMMUNE SUPPRESSION	OMIM:146850	9.28	2
SOCS3	Liver Diseases	MESH:D008107	189.83	133
	Chemical and Drug Induced Liver Injury	MESH:D056486	333.77	1474
	Liver Failure, Acute	MESH:D017114	75.42	207
	Liver Failure	MESH:D017093	36.17	35
	Bacterial Infections	MESH:D001424	30.53	11
	Infections	MESH:D007239	15.96	5
	Bacteremia	MESH:D016470	2.06	2
	Sepsis	MESH:D018805	53.29	52
	Immune System Diseases	MESH:D007154	66.44	21
	IMMUNE SUPPRESSION	OMIM:146850	2.8	1

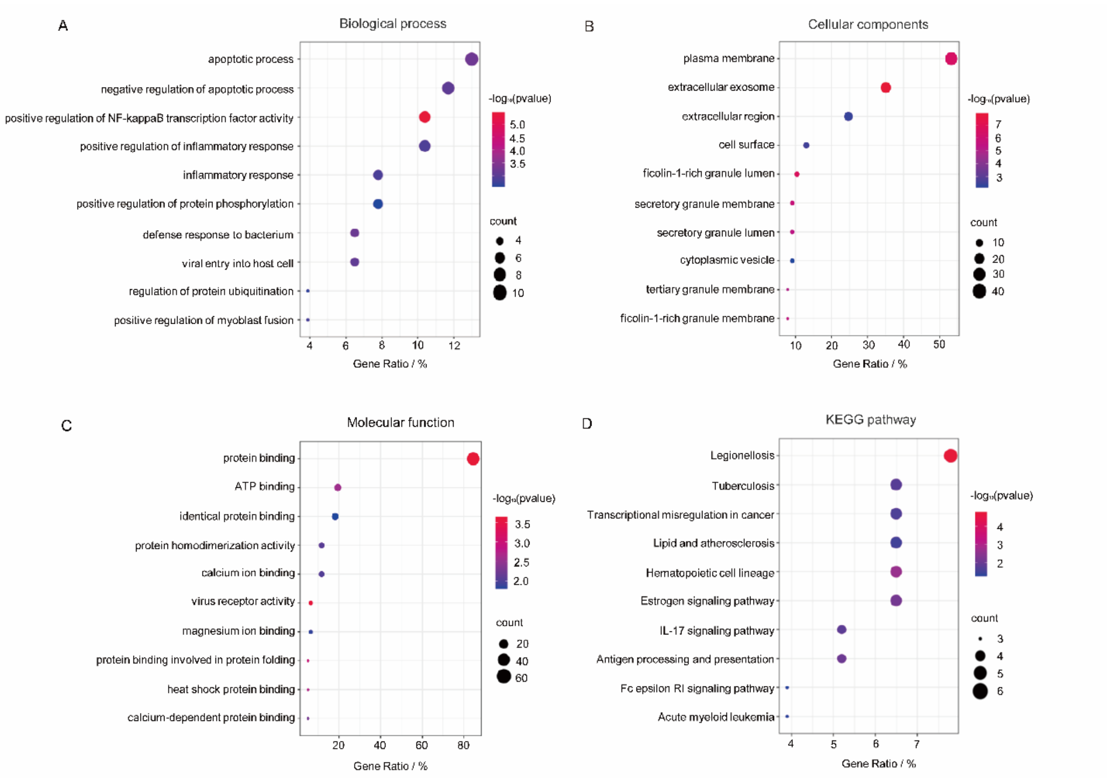

Figure S1. Functional analysis of common DEGs in human datasets.

(A–D) Top 10 shared DEGs based on BP, CC, MF, and KEGG analysis in the human dataset.

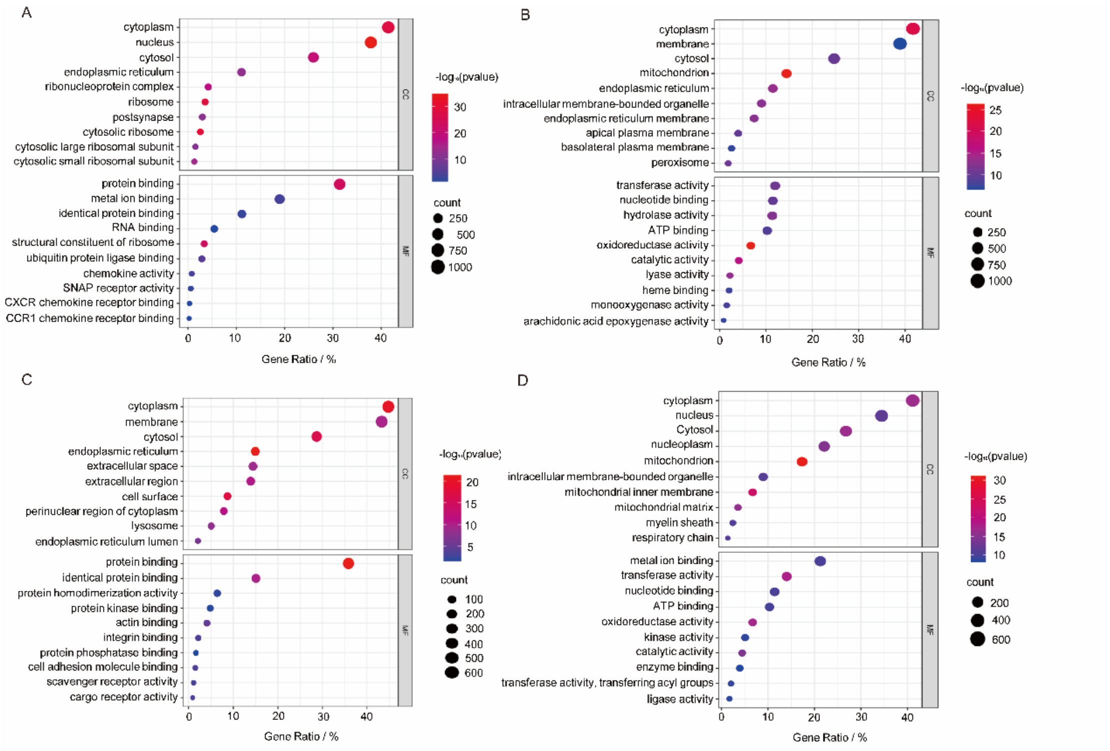

Figure S2. Functional analysis of DEGs in different mouse models.

(A–B) The top 10 upregulated and downregulated genes based on CC and MF analysis in the GSE184167 dataset. (C–D) The top 10 upregulated and downregulated genes based on CC and MF analysis in the GSE166488 dataset.

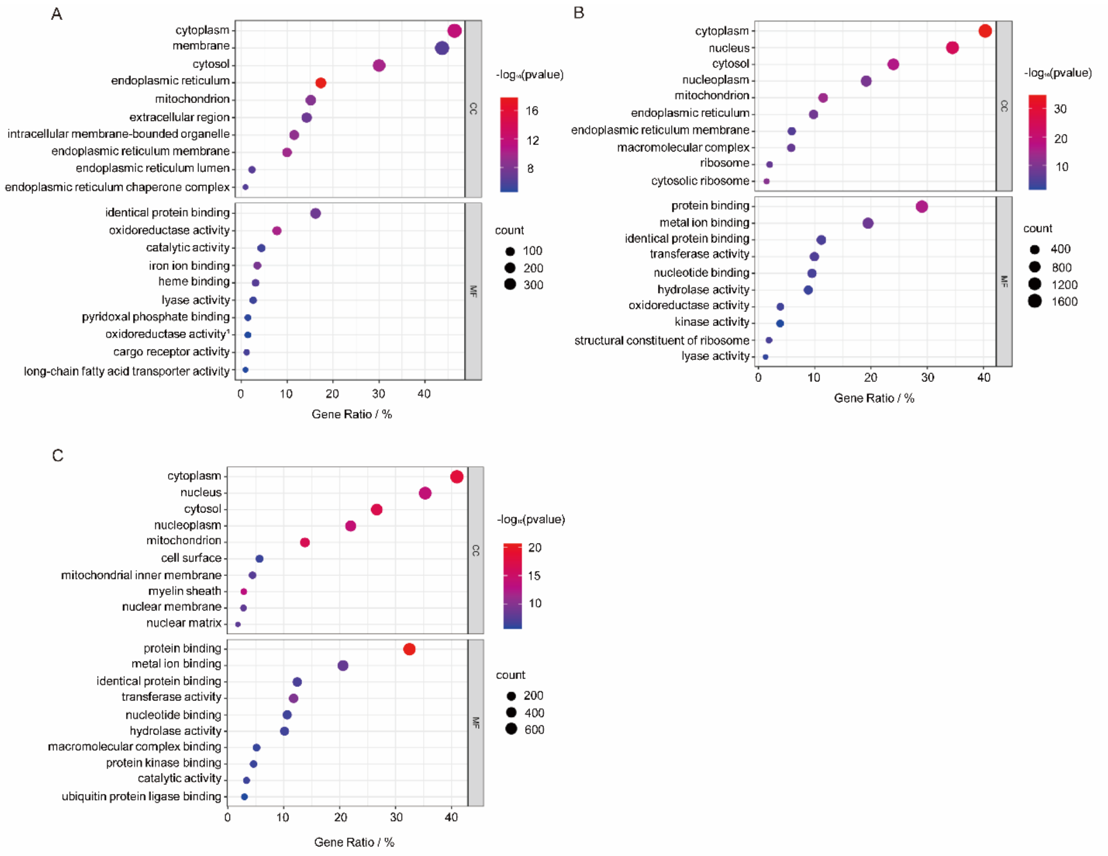

Figure S3. Functional analysis of DEGs in human sepsis and different mouse sepsis models.

(A) The top 10 DEGs in the intersection of the CLP mouse model and the LPS mouse model based on CC analysis and MF analysis. (B–C) The top 10 DEGs specific to the CLP group and LPS group based on CC analysis and MF analysis. Note: Oxidoreductase activity[1] in Figure A represents oxidoreductase activity, acting on paired donors, with incorporation or reduction of molecular oxygen, reduced flavin or flavoprotein as one donor, and incorporation of one atom of oxygen.

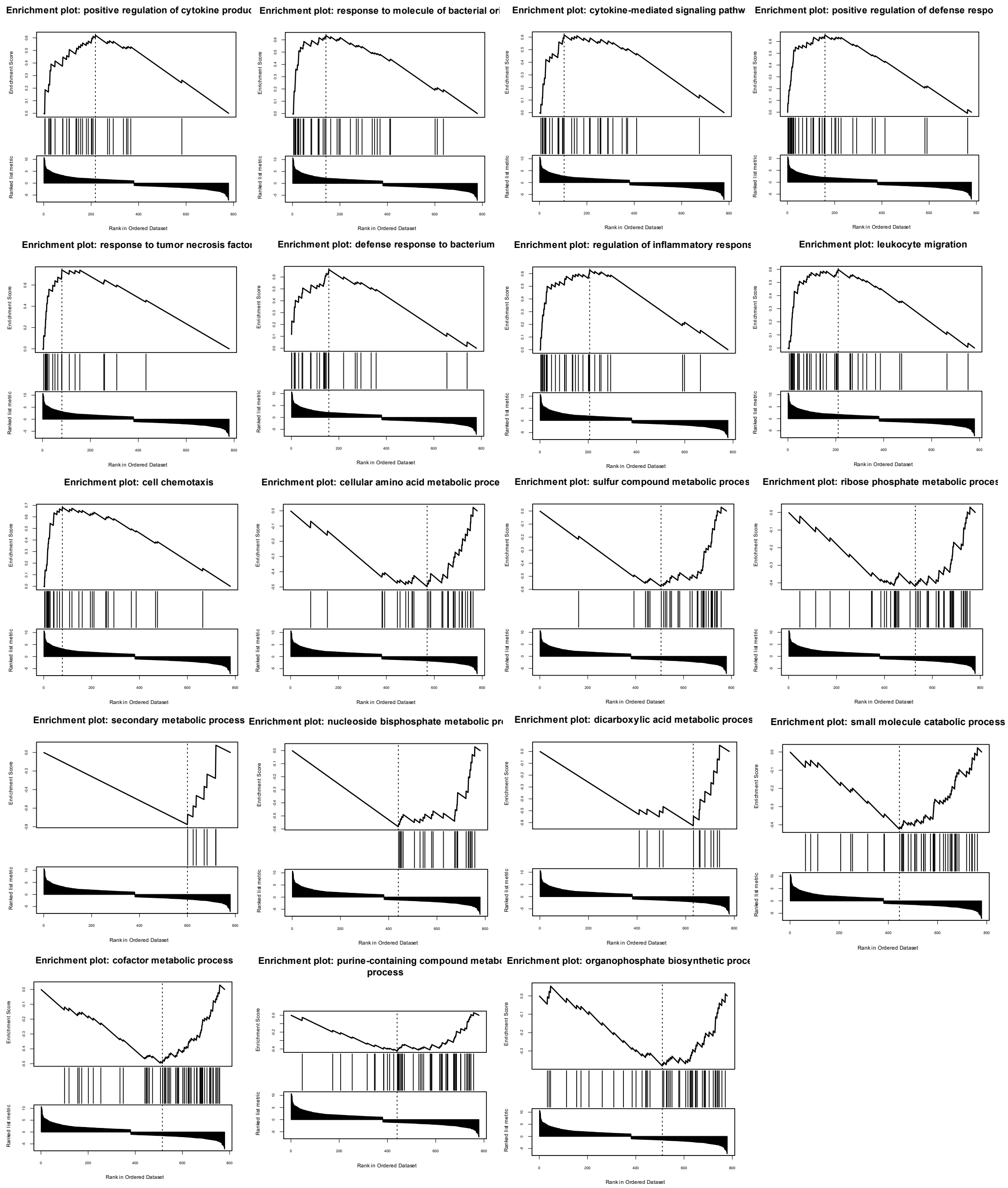

Figure S4. GSEA enrichment analysis of mouse CLP model and LPS model intersecting genes in CLP group

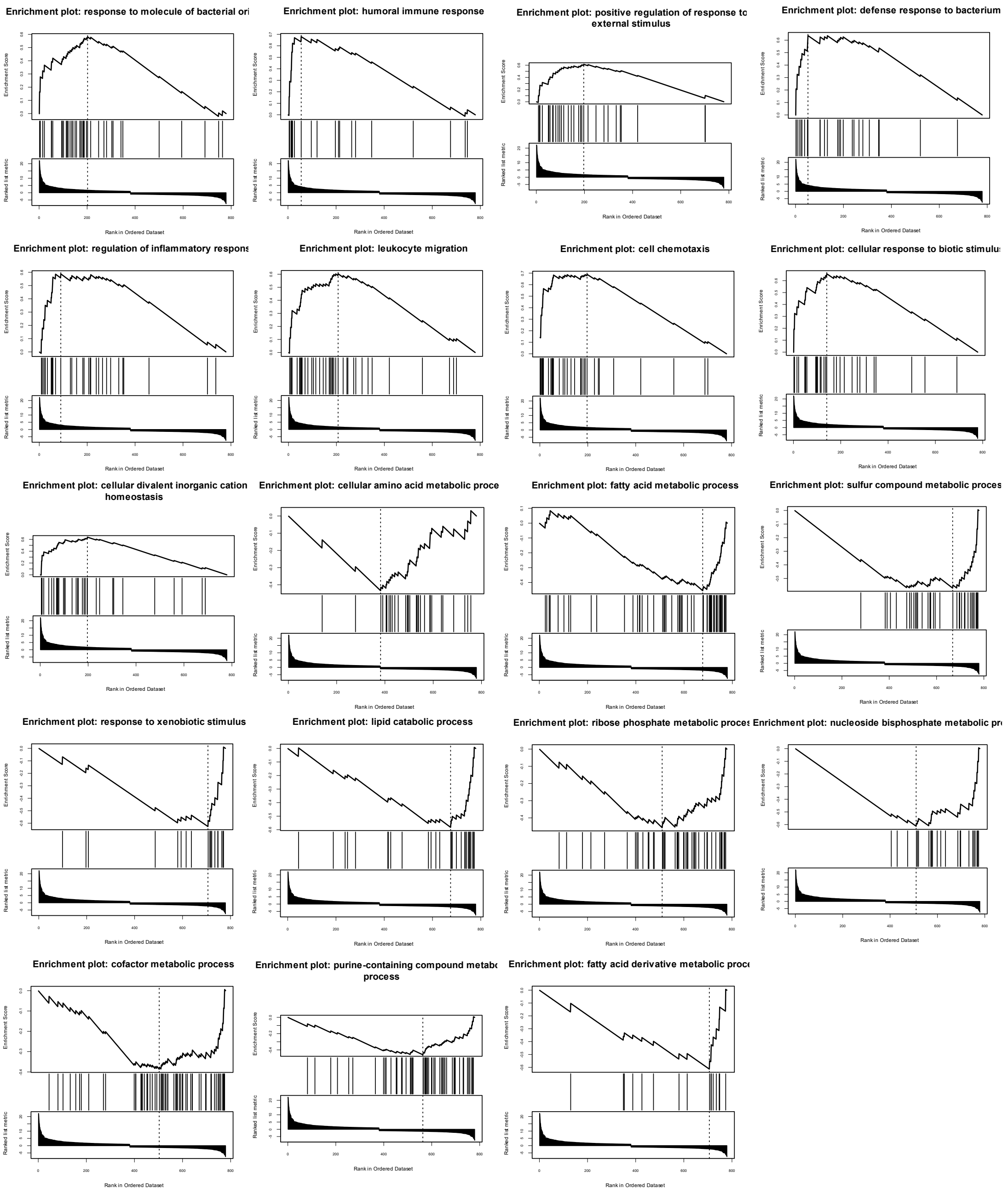

Figure S5. GSEA enrichment analysis of mouse CLP model and LPS model intersecting genes in LPS group.

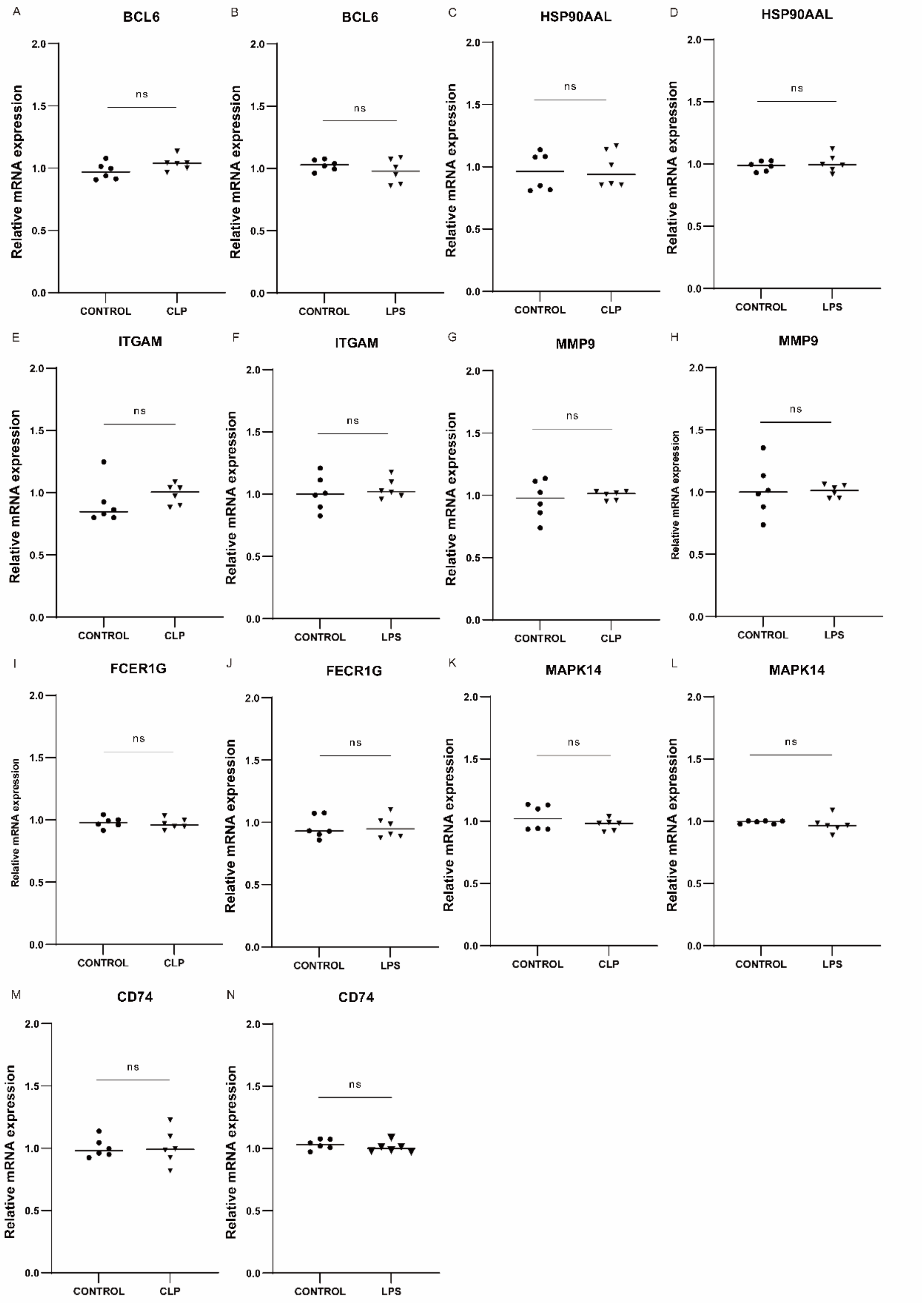

Figure S6. Relative RNA expression of core DEGs.
(A–J) Relative RNA expression of *CD44,MAPK14,CD74,HSP90AAL,NLRC4* in the CLP group and LPS group.
